# The difficulty of translating “well-being” from English to Arabic

**DOI:** 10.2471/BLT.24.293044

**Published:** 2025-06-01

**Authors:** Kinda Alsamara, David Forbes

**Affiliations:** a School of Languages and Cultures, University of Queensland, Brisbane, QLD 4072, Australia.; b Maudsley Health, Abu Dhabi, United Arab Emirates.

Since the launch of the World Health Organization (WHO) Mental Health Global Action Programme in 2002, addressing the mental health crisis in disadvantaged populations has become a priority for international health organizations. This focus has been accompanied by investment into global public health initiatives and interventions, some of which have targeted the Arabic-speaking countries across the Eastern Mediterranean Region, whose populations are experiencing acute and persisting humanitarian crises.^1^ The domain of mental health has traditionally been stigmatized across the Arabic-speaking world.^2^ Consequently, these global public health campaigns have focused on reducing this stigma, increasing awareness and acceptance of mental health and promoting engagement in related initiatives. Most importantly, these initiatives have also sought to extend the focus on mental health to include well-being as a fundamental right – and not what might be perceived as a privilege in the eyes of disadvantaged communities.^1^ A notable example is the WHO framework document *Achieving well-being: a global framework for integrating well-being into public health utilizing a health promotion approach*.^1^ Unfortunately, the word used for well-being in this document is *Rafāh*. While other international bodies also use *Rafāh* for well-being, here we explain why we believe the use of this word is ill-advised.

Many international policy and public health initiatives mistranslate the term “well-being” in their Arabic translations and public health communications as *Rafāh* or *Rafāhīyah*.^3^ In reviewing major Arabic dictionaries, including *al-Mu´jam al-Wasīt *and *Lisān al-´Arab,* the word *Rafāhīyah* is clearly defined as luxury. *Rafāhīyah* is also the technical name for a formal government luxury tax on luxury items. In the absence of a word for well-being in Arabic, translators in international bodies unilaterally determined and adopted the word *Rafāh* as a new word to use for well-being.^4^ However, its root is the same as *Rafāhīyah*, and using this new word with no history but with a default interpretation referring to luxury is a considerable risk.^4^

In the UN Secretary-General’s 2023 statement, the phrase “Human rights are not a luxury that can be postponed until we solve the world’s other problems” was translated into Arabic using *Rafāhīyah* for the word luxury.^5^ This translation recognizes its understood meaning. However, UN communications then use this same translated term, *Rafāhīyah,* to mean well-being in other reports.^3^ When UN translators use this same term for both concepts, misunderstandings are almost inevitable, preventing the intended message from effectively reaching communities where such concepts already carry significant stigma. Some Arabic-speaking governments and translation bodies have sought to bypass the confusion and avoid referring to either *Rafāh* or *Rafāhīyah* by using *Jawdat al-Hayāt,* the Arabic words for quality of life, when referring to well-being. However, while this solution is creative and more helpful than the alternative, quality of life and well-being are not the same.

WHO and other international global bodies invest considerable effort to reduce the stigma of mental health in the Arabic-speaking world. While this issue can appear semantic, the translation of well-being using a word that means luxury potentially disenfranchises a population that already has barriers to accepting mental health and well-being as a right, not a privilege. 

The absence of a genuinely and culturally derived Arabic word for well-being is a product of the very gap that international health and UN-affiliated institutions have attempted to address. However, rather than a potentially counterproductive, unilaterally determined mistranslation, perhaps an opportunity exists for a WHO-led initiative to engage with the Arabic-speaking world in a systematic co-design process to identify a word or phrase that is meaningful, acceptable and culturally resonant. While systematic attention might be needed, the results have the potential to increase the accessibility and acceptability of the idea of well-being across the Arabic-speaking world. Furthermore, such an initiative would advance the potential to optimize the considerable investment in remediating stigma, reducing barriers to mental health and well-being, and increasing access of services and initiatives provided to those most in need. Such a co-design process is also the start of a culturally respectful approach to combating the stigma in its own right. The choice of a word is far more than semantics.
